# Transcriptional responses define dysregulated immune activation in Hepatitis C (HCV)-naïve recipients of HCV-infected donor kidneys

**DOI:** 10.1371/journal.pone.0280602

**Published:** 2023-01-26

**Authors:** Julie M. Steinbrink, Cameron Miller, Rachel A. Myers, Scott Sanoff, Anna Mazur, Thomas W. Burke, Jennifer Byrns, Annette M. Jackson, Xunrong Luo, Micah T. McClain

**Affiliations:** 1 Division of Infectious Diseases, Department of Medicine, Duke University Medical Center, Durham, NC, United States of America; 2 Center for Applied Genomics & Precision Medicine, Department of Medicine, Duke University, Durham, NC, United States of America; 3 Division of Nephrology, Department of Medicine, Duke University Medical Center, Durham, NC, United States of America; 4 Department of Pharmacy, Duke University Medical Center, Durham, NC, United States of America; 5 Departments of Surgery and Immunology, Duke University, Durham, NC, United States of America; 6 Division of Infectious Diseases, Durham Veterans Affairs Health Care System, Durham, NC, United States of America; University of Cincinnati College of Medicine, UNITED STATES

## Abstract

Renal transplantation from hepatitis C (HCV) nucleic acid amplification test-positive (NAAT-positive) donors to uninfected recipients has greatly increased the organ donation pool. However, there is concern for adverse outcomes in these recipients due to dysregulated immunologic activation secondary to active inflammation from acute viremia at the time of transplantation. This includes increased rates of cytomegalovirus (CMV) DNAemia and allograft rejection. In this study, we evaluate transcriptional responses in circulating leukocytes to define the character, timing, and resolution of this immune dysregulation and assess for biomarkers of adverse outcomes in transplant patients. We enrolled 67 renal transplant recipients (30 controls, 37 HCV recipients) and performed RNA sequencing on serial samples from one, 3-, and 6-months post-transplant. CMV DNAemia and allograft rejection outcomes were measured. Least absolute shrinkage and selection operator was utilized to develop gene expression classifiers predictive of clinical outcomes. Acute HCV incited a marked transcriptomic response in circulating leukocytes of renal transplant recipients in the acute post-transplant setting, despite the presence of immunosuppression, with 109 genes significantly differentially expressed compared to controls. These HCV infection-associated genes were reflective of antiviral immune pathways and generally resolved by the 3-month timepoint after sustained viral response (SVR) for HCV. Differential gene expression was also noted from patients who developed CMV DNAemia or allograft rejection compared to those who did not, although transcriptomic classifiers could not accurately predict these outcomes, likely due to sample size and variable time-to-event. Acute HCV infection incites evidence of immune activation and canonical antiviral responses in the human host even in the presence of systemic immunosuppression. After treatment of HCV with antiviral therapy and subsequent aviremia, this immune activation resolves. Changes in gene expression patterns in circulating leukocytes are associated with some clinical outcomes, although larger studies are needed to develop accurate predictive classifiers of these events.

## Introduction

There is a large disparity between the available number of kidney organ donors and recipients on the transplant waitlist–patients spend years on hemodialysis waiting for a kidney. Hepatitis C (HCV) nucleic acid antigen positive (NAAT-positive) donors are therefore being transplanted at a rapidly escalating rate to uninfected recipients to increase organ availability. This is due to the significant advancements made in the development of direct acting antiviral medications for the treatment and cure of HCV. These medications offer many benefits including high potency, low side effect profile, and pan-genotypic coverage [1–3].

However, there is concern that active HCV viremia results in an inflammatory state at the time of transplant and this may have downstream immunologic effects on the transplant recipient. The full extent of such effects is still being clarified. There have been reports of increased incidence of adverse outcomes in HCV transplant recipients, including increased incidence of *de novo* donor specific antibodies (DSA), allograft rejection, and reactivation of opportunistic viral infections, such as cytomegalovirus (CMV) [4–7]. These inflammatory events can be more closely examined by reviewing the host transcriptomic response in peripheral blood.

Immune activation can be measured and monitored in the peripheral blood at the transcriptomic level in the transplant recipient. It is known that chronic HCV viremia triggers unique transcriptomic and cytokine responses that clarify disease diagnosis, pathophysiology, and severity [8–10]. This infection triggers an inflammatory state, with increased cytokine production and the expression of genes involved in the antiviral response, namely interferon. Additionally, genes involved in the clearance of HCV viremia have unsurprisingly been identified to primarily involve those related to antiviral immune responses, while genes involved in the interferon-mediated antiviral response are not notably upregulated in patients who developed progressive disease due to HCV, including early liver fibrosis [11]. Prior studies, however, have primarily investigated the timeline to resolution of these viral signals in non-immunocompromised subjects with acute and chronic infection, and have not explored the discrepancies that may arise in immunocompromised patients, such as transplant recipients [8, 12–16].

Gene expression profiling has also been used to noninvasively detect rejection in solid organ transplantation [17–23]. However, there remains little known about the interplay between HCV infection-induced immune activation and this important outcome. In order to examine these issues, we performed serial sampling and transcriptomic analysis of the peripheral blood of renal transplant recipients with and without HCV-NAAT-positive donors and assessed the relationship between particular gene expression patterns and specific clinical outcomes–namely CMV DNAemia and allograft rejection.

## Materials and methods

### Subject enrollment and sampling

All study patients were enrolled after written informed consent at Duke University Medical Center (DUMC). The study was approved by the Institutional Review Board (IRB) at DUMC (Pro00106012) and was performed in accordance with the Declaration of Helsinki. Sixty-seven patients without HCV infection who underwent renal transplantation from July 2020 to August 2021 were enrolled at the time of renal transplantation, including subjects who received an organ from a donor who was HCV-NAAT-positive, as well as subjects who received an organ from a donor who was HCV-NAAT-negative (Controls).

Whole blood was collected from all subjects in PAXgene Blood RNA Tubes (QIAGEN) for RNA sequencing and serum was collected for additional analysis. Each subject had planned sample collections at the first initial outpatient Infectious Diseases clinic visit just prior to the start of antiviral therapy (or corresponding timepoint for controls) at a median of 17 days post-transplant (IQR 15–22) (Timepoint 1); at the end of 12-weeks of antiviral therapy (approximately 3 months post-transplant, Timepoint 2); and at a sustained virologic response 12 weeks after treatment (SVR12) (12 weeks after the end of antiviral therapy, approximately 6 months post-transplant, Timepoint 3). DSA testing was performed using multianalyte bead assays (LABScreen-Single Antigen and FlowPRA, One Lambda) for all subjects at 6 months post-transplant.

All HCV-NAAT-positive organ recipients were treated with 12 weeks of a direct-acting antiviral regimen–either ledipasvir-sofosbuvir, sofosbuvir-velpatasvir, or glecaprevir-pibrentasvir, per standard of care transplant management. Treatment regimens were chosen by the patient’s primary Transplant Infectious Diseases provider independently of the study, based on patient comorbidities, medication interactions, and insurance coverage. All antiviral regimens were started in the outpatient setting after index transplant hospitalization.

### Definitions of CMV DNAemia and rejection

At DUMC, low-risk CMV patients (donor negative/recipient negative, or D-/R-) are treated with universal prophylaxis for herpes simplex virus (HSV) with acyclovir only. In intermediate risk populations (R+), renal transplant recipients use pre-emptive prophylaxis with acyclovir for 90 days with weekly CMV polymerase chain reaction (PCR) monitoring if they received steroid induction, or universal prophylaxis for 180 days with valganciclovir if they received thymoglobulin. High risk CMV populations (D+/R-) are treated with universal CMV prophylaxis with valganciclovir for at least 180 days.

CMV DNAemia was detected and quantified using quantitative PCR (COBAS^®^ AmpliPrep/COBAS^®^ TaqMan^®^ CMV Test at DUMC). Patients with “clinical” CMV DNAemia were defined as those with equal or greater than 137 international units/milliliter (IU/mL) of CMV DNA present (or equivalent external lab). Patients with “subclinical” CMV infection were defined as those that had detectable CMV DNA below 137 IU/mL (the lower limit of quantification of the PCR). Lastly, those patients with no detectable CMV DNA were termed the “no CMV” group.

Analyses were performed comparing either (1) patients with any CMV infection (composed of patients with both subclinical infection and clinical CMV) to patients with no CMV infection or (2) clinical CMV patients to patients with no CMV. For the comparison in (2), patients with subclinical CMV infection (less than 137 IU/mL) were excluded from analysis.

Rejection was defined as both biopsy-proven allograft cellular rejection and the development of DSAs post-transplant in order to capture both cellular and antibody-mediated rejection outcomes.

### Sequencing

Upon collection, whole blood was stored at -80° Celsius (C) in PAXgene Blood RNA tubes. Total RNA was isolated and purified using the QIAGEN PAXgene Blood miRNA kit. Stranded cDNA libraries were prepared using the Nugen Universal Plus mRNA-seq kit with Globin AnyDeplete (Tecan Life Sciences) yielding stranded, poly(A)-selected, globin-depleted cDNA sequencing libraries. The cDNA libraries were sequenced by the Sequencing and Genomic Technologies Shared Resource within the Duke Center for Genomic and Computational Biology in two batches, using an Illumina NovaSeq 6000 instrument that generated 50bp paired-end reads. Sequences were aligned and quantified with Spliced Transcripts Alignment to a Reference (STAR), using the NCBI Homo sapiens GRCh38 genome as a reference [24].

### Data processing

Following sequencing, alignment, and quantification, all subsequent data processing and analyses were performed using R [25]. Initial multi-dimensional scaling and principal components analysis revealed significant batch effects between the two sequencing batches. Therefore, lowly expressed transcripts were filtered separately for the batches, and then the batches were combined prior to normalization. For Batch 1, transcripts that exceeded 2.25 counts per million (CPM) in at least 70% of samples were kept. For Batch 2, transcripts that exceeded 1 CPM in at least 65% of samples were kept. The filtered transcripts across batches were combined, and the expression data were normalized using the trimmed mean of M-values (TMM), available in the edgeR package [26].

### Comparison of clinical demographics

Comparison of clinical demographics was performed by chi-square test for categorical variables or Mann-Whitney for continuous variables.

### Differential expression analysis

Differential expression analysis (DEA) was carried out using the limma package within R [27]. A series of linear models was fit to the expression data, one transcript at a time. For a list of the models fit, see S1 Table in S1 File. All models included dummy variables for the RNA extraction batch in order to account for technical effects. The RNA extraction batch was nested within the sequencing batch, and so the sequencing batch was not included as a covariate. Models that included sampling from multiple timepoints accounted for within-patient correlation in the following way. Observation-level voom weights were generated by estimating the log_2_-transformed mean-variance relationship. The within-patient correlation was estimated given the weights, and then the weights were recalculated using the correlation estimate. Upon fitting the linear models, p-values were adjusted using the false discovery rate (FDR) using the Benjamini-Hochberg method, and transcripts with an FDR ≤5% were considered differentially expressed.

### Gene set enrichment analysis (GSEA)

GSEA [28]. was performed using the clusterProfiler R package [29]. Three ontologies within the gene ontology (GO) gene sets were searched for enrichment: Biological Process (BP), Cellular Component (CC), and Molecular Function (MF). Only those gene sets that had at least 10 genes but not more than 1,000 were searched. The p-values were adjusted for multiple testing using the FDR, and gene sets with an FDR ≤5% were considered significantly enriched.

### Predictive analysis

Least absolute shrinkage and selection operator (LASSO), available in the R package glmnet [30], was used to assess the ability of the transcriptomics to predict any CMV DNAemia or allograft rejection. First, among all transcripts, the 1,000 with the highest variance in expression were selected to build predictive models. All subsequent models contained those 1,000 transcripts, as well as the RNA extraction batch. The RNA extraction batch was nested wholly within the sequencing batch, meaning all samples in an extraction batch were sequenced in a single batch. Therefore, to account for technical effects, dummy variables for the RNA extraction batches were included, although they were not subject to regularization and so remained in every model fit.

One hundred iterations of the following procedure were performed. The data (transcriptomics and clinical) were subsampled so that for cases the most recent blood sample prior to the outcome was utilized. For CMV DNAemia, this meant the most recent sample prior to the detection of CMV. Any patient with samples taken only after detection of CMV DNAemia was removed from the corresponding analysis. For controls, a single, randomly selected blood sample was used. One-by-one each observation was held out, and three-fold cross-validation (CV) was used on the remaining observations to train a LASSO model. The training model was optimized in order to minimize the deviance, the probability of the corresponding outcome for the held-out observation was predicted using the tuning parameter value that minimized the deviance.

### Association analysis

These analyses focused on identifying transcripts associated with outcomes of interest and not on building prediction models. As with the predictive analysis, the 1,000 transcripts with the highest variance in expression were selected to build a predictive model. All subsequent models contained those 1,000 transcripts, as well as the RNA extraction batch.

For each outcome, 500 iterations of a leave-one-out cross-validation (LOOCV) procedure were performed. As with the predictive analysis, the data were subsampled such that, for cases, the most recent blood sample prior to the corresponding outcome was used, and for controls a randomly sampled blood sample was used. LOOCV was used to select a value for the LASSO regression tuning parameter that minimized the deviance, and the coefficients corresponding to that tuning parameter were extracted to represent the final fit for that iteration.

### Comparison with publicly available datasets

The transcriptomic signals for CMV DNAemia (excluding subclinical infection) and rejection were compared with those from publicly available datasets. For CMV DNAemia, we utilized a case-control study examining longitudinal changes in expression upon the onset of DNAemia, defined as CMV DNA concentrations >137 IU/mL (GEO Accession Number: GSE168598) [31]. This study from Ahn, et al. sampled control patients once and then cases prior to DNAemia and again at three timepoints following DNAemia: 1 week, 1 month, and long-term (approximately 1 year). Our study did not examine changes in gene expression beyond 6 months post-transplant, so the long-term timepoint was removed from the Ahn, et al. dataset.

We compared transcriptomic signals in our study associated with rejection with a prospective study focused on finding noninvasive biomarkers for acute cellular rejection [18]. The comparative study contained discovery and validation datasets, composed of expression data taken at 3 months following kidney transplant. At the same 3-month timepoint the clinical endpoint of acute cellular rejection was determined by kidney biopsy.

The results from DEA and GSEA were compared across studies. For DEA, the log_2_-transformed counts per million for all transcripts were regressed on the outcomes using the R package limma, and p-values were adjusted for multiple testing using the FDR. The lists of transcripts exceeding different significance thresholds were compared. Furthermore, the log_2_ fold-changes and p-values for both comparative studies were plotted simultaneously to look for gross similarities.

## Results

### Study population and clinical outcomes

We enrolled 67 subjects following renal transplantation– 37 recipients of kidneys from HCV-NAAT-positive organ donors and 30 recipients of kidneys from HCV-NAAT-negative organ donors (Controls). Full demographics are described in Table 1. Not all subjects were able to be sampled at all timepoints– 40 subjects had PAXgene Blood RNA samples at Timepoint 2 (18 controls, 22 HCV-infected), while 31 were sampled at Timepoint 3 (15 controls, 16 HCV-infected). Induction immunosuppression regimens were similar between the HCV-infected recipients and controls–this consisted primarily of steroid induction, with escalation to thymoglobulin in the setting of high-risk recipient characteristics or delayed graft function. Maintenance immunosuppression was also similar between the two groups and consisted of an antimetabolite, a calcineurin inhibitor, and a steroid. All HCV-NAAT-positive organ recipients were treated with 12 weeks of a direct acting antiviral regimen–either ledipasvir-sofosbuvir, sofosbuvir-velpatasvir, or glecaprevir-pibrentasvir. All antiviral regimens were started in the outpatient setting after index transplant hospitalization, at an average of 36 days (range 11–80 days) post-transplant.

**Table 1:** Clinical demographics of all enrolled subjects.

Demographics	HCV-Donor (n = 37)	Control (n = 30)	P-value
**Female**	12	14	0.235
**Mean Age (years) ± SD**	56.31 ± 11.05	58.59 ± 11.27	0.335
**Race**			
** American Indian/Alaskan Native**	0	0	
** Asian**	1	0	0.364
** Black/African American**	23	11	0.038
** Native Hawaiian/Pacific Islander**	0	0	
** White**	14	16	0.2047
** Unknown**	0	2	0.111
** Multiracial**	1 (Asian and White)	1	0.880
**Prior Transplant**	4	7	0.199
** Heart**	2	2	
** Kidney**	1	2	
** Liver**	0	3	
** Lung**	1	0	
** Multiorgan**	0	0	
**Multi-organ Transplant**	2	4	0.396
** Heart**	0	1	
** Liver**	1	2	
** Lung**	1	0	
** Pancreas**	0	1	
**Induction Immunosuppression**			
** Alemtuzumab**	1	0	0.364
** Basiliximab and steroids**	2	1	0.683
** Steroids only**	18	18	0.354
** Steroids and Thymoglobulin**	7	3	0.308
** Thymoglobulin**	9	8	0.827
**Genotype**		NA	
** 1a**	19		
** 1b**	3		
** 2**	4		
** 3**	10		
** 4**	1		
**Initial HCV Treatment**		NA	
** Glecaprevir-pibrentasvir**	20		
** Ledipasvir-sofosbuvir**	5		
** Sofosbuvir-velpatasvir**	12		

SD = standard deviation

**Fig 1:**
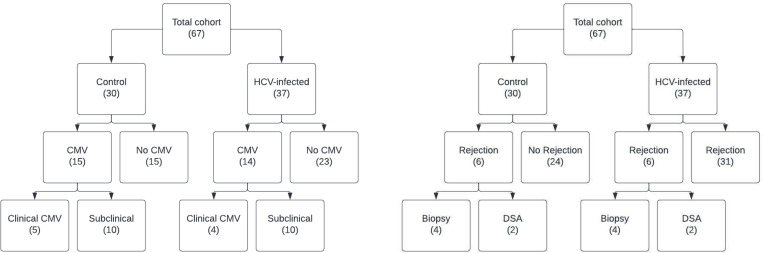
Cohort diagram. Cohort diagram of all enrolled study participants, with breakdown into CMV (left) and rejection (right) subgroups.

CMV DNAemia was defined as any detectable level of CMV on quantitative PCR from peripheral blood. Twenty-nine subjects had evidence of CMV DNAemia during the study period (viral load range <137 to 59,700 IU/mL) (Fig 1). These consisted of 15 control subjects and 14 HCV-infected subjects (S2 Table in S1 File). “Subclinical” CMV DNAemia was defined as any CMV DNA detectable but less than 137 IU/mL (20 subjects: 10 controls, 10 HCV-infected). “Clinical” CMV DNAemia was defined as any CMV DNA greater than 137 IU/mL (9 subjects: 5 controls, 4 HCV-infected). No organ recipients had evidence of tissue-invasive CMV disease. Time from transplant to peak DNAemia was a median of 58 days (IQR 22 to 102 days)–this was 58 days for the controls (IQR 17 to 67); compared to 55 days for the HCV cohort (IQR 31 to 136) (p = 0.394).

Twelve subjects demonstrated evidence of allograft rejection– 6 controls and 6 HCV-infected recipients (Fig 1). Rejection was defined as both biopsy-proven allograft cellular rejection and the development of *de novo* DSAs post-transplant– 8 subjects demonstrated evidence of acute cellular rejection (4 control subjects and 4 with HCV-infected donors) while 4 recipients had evidence of positive DSAs at approximately 6 months post-transplant (2 control subjects and 2 with HCV-infected donors) (S3 and S4 Tables in S1 File). The median time from transplant to detection of total rejection episodes was 215 days (IQR 94 to 261)–for controls, this was 200 days (IQR 55 to 267); for the HCV cohort this was 215 days (IQR 110 to 238) (p >0.999).

### Acute HCV sparks a robust transcriptomic response in renal transplant recipients that resolves over time with antiviral therapy

**Fig 2:**
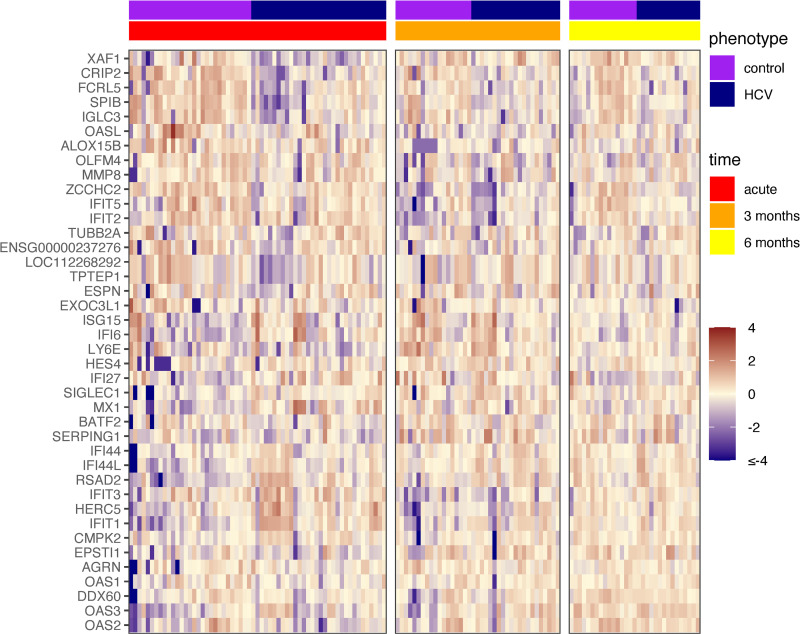
Heatmap of HCV vs control samples at acute, 3-month, and 6-month timepoints. The list along the y-axis includes all transcripts with |log fold changes| ≥ 1 comparing HCV and control samples. Gene names are sorted using a clustering algorithm to group the genes based on expression, combining those that are most similar. Differences in gene expression are noted at the acute timepoint between HCV and control samples, that decrease and resolve by the later timepoints.

We first performed a univariate analysis of HCV-NAAT-positive organ recipients compared to controls. We examined differential gene expression across all enrolled subjects at the acute, 3-month, and 6-month post-transplant timepoints. We found that acute HCV infection sparked a robust transcriptomic response with 109 differentially expressed genes compared to controls, even in the setting of systemic immunosuppression (Fig 2). These findings were most apparent at the acute timepoint, approximately 17 days post-transplantation. This consisted of genes known to be involved in the antiviral response, including interferon-induced proteins (IFITs), interferon stimulated genes (ISGs), and oligoadenylate synthetase-like genes (OASLs).

In order to examine the biological implications of gene expression patterns associated with acute post-transplant HCV infection, we also analyzed gene lists for significantly enriched pathways at early post-transplant times. Many of the genes identified had been previously reported specifically in the host response to acute HCV infection in the absence of immunosuppression [16]. These pathways included biological processes such as the ‘defense response to virus,’ ‘cellular response to virus,’ ‘innate immune response,’ ‘regulation of interferon-beta production,’ ‘type I interferon-mediated signaling,’ ‘cellular response to interferon alpha,’ ‘interleukin-mediated signaling,’ and ‘activation of the innate immune response.’ These genes were no longer significantly differentially expressed between HCV and control cohorts at the 3- and 6-month timepoints post-transplant following HCV treatment and aviremia (Fig 2).

### Gene expression differences amongst transplant recipients who develop CMV DNAemia

Given fixed sample collection times, samples were often not available at or near the time of an event of interest (i.e., CMV DNAemia). The median time from sample collection to first detected CMV DNAemia (including subclinical CMV, for samples collected prior to the outcome only) was -48 days (IQR -26 to -139). This was -47 days (IQR -25 to -89) for controls and -84 days for the HCV cohort (IQR -34 to -129) (p = 0.680). The median time from sample collection to clinical CMV DNAemia (excluding subclinical CMV, for samples prior to the outcome only) was -49 days (IQR -26 to -92). This was -67 days for controls (IQR -44 to -90) and -42 days for the HCV cohort (IQR -23 to -106) (p = 0.558).

To better understand the relationship between gene expression differences and specific adverse transplant clinical outcomes, we performed univariate analyses of subjects who developed CMV DNAemia post-transplant compared to those who did not, both in the presence and absence of HCV. To assess for the presence of a persistently dysregulated transcriptomic signal that begins prior to CMV DNAemia, continues to be present over time, and is conserved both in the presence and absence of HCV, we first reviewed differential gene expression data in all subjects at all timepoints within each group (all CMV vs no CMV). In the transplant recipients who developed CMV DNAemia (both subclinical and clinical, n = 29 subjects), 995 genes were upregulated and 974 genes were downregulated, compared to those who did not develop CMV. When the CMV DNAemia cutoff was increased to >137 IU/mL (clinical CMV, n = 9 subjects), the number of upregulated genes increased to 1,187, while 808 genes were downregulated. This included genes involved in immune activation and T cell signaling, including biological processes such as ‘T cell immunoreceptor,’ ‘T cell receptor gamma,’ ‘Fc receptor,’ and ‘lymphocyte activation.’ 335 of those genes overlapped with published CMV response patterns in renal transplant recipients [31].

To identify specific HCV-associated differences in the immune response to CMV infection, we next examined the differential gene expression data of transplant recipients who developed CMV DNAemia (all samples, all timepoints, including subclinical CMV) in the control cohort (n = 15 subjects) compared to the HCV-infected cohort (n = 14 subjects). In the control group, 52 genes were upregulated and 53 were downregulated. In the HCV cohort, this number was much lower, with only 5 genes upregulated and 1 downregulated. We inspected these gene expression differences separately at each individual post-transplant timepoint. However, in both the HCV and control groups, no genes were significantly differentially expressed at any timepoint.

#### Gene expression changes predictive of CMV DNAemia

**Fig 3:**
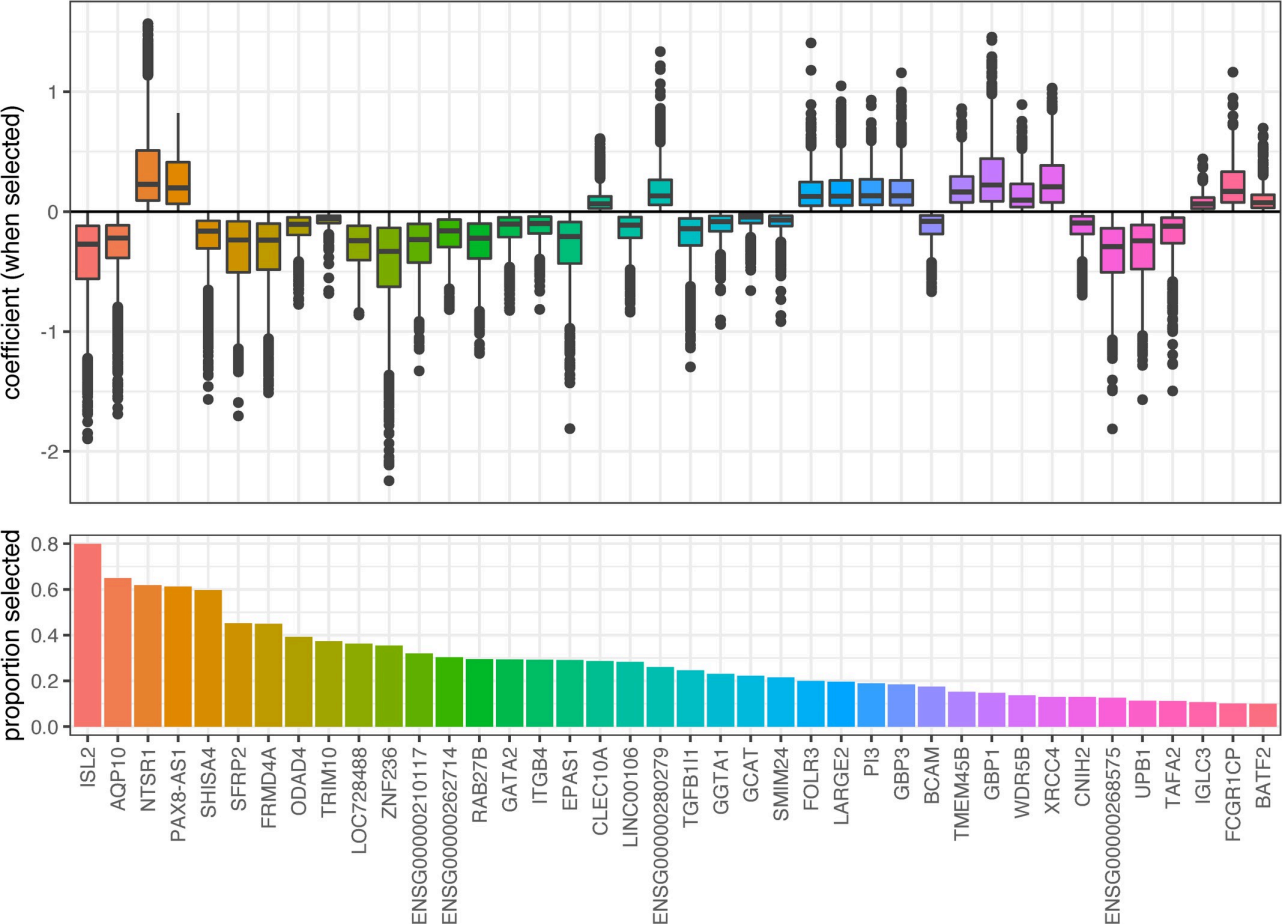
LASSO regression training results for the prediction of any CMV DNAemia. For each of 100 iterations, 57 models were trained to predict any CMV DNAemia. The bottom panel shows the proportion of all fits that selected each gene or transcript, and the top panel shows boxplots of the coefficients, when the corresponding terms were selected for inclusion. The genes and transcripts shown are those that were selected in 10% or more models. They are sorted in decreasing order of selection frequency, and are labeled with their gene symbol when available or the Ensembl ID.

We next examined the data for transcriptomic signatures that are predictive of CMV DNAemia after transplantation. A LASSO regression model was repeatedly fit to samples of the original data. For patients that exhibited CMV DNAemia (clinical and subclinical), the blood sample taken prior to the detection of CMV was used, and for patients that did not exhibit any CMV DNAemia, a random blood sample was used. Patients with no blood samples prior to detection of CMV were removed from the analysis. The transcripts selected in the predictive modeling are summarized in Fig 3.

**Fig 4:**
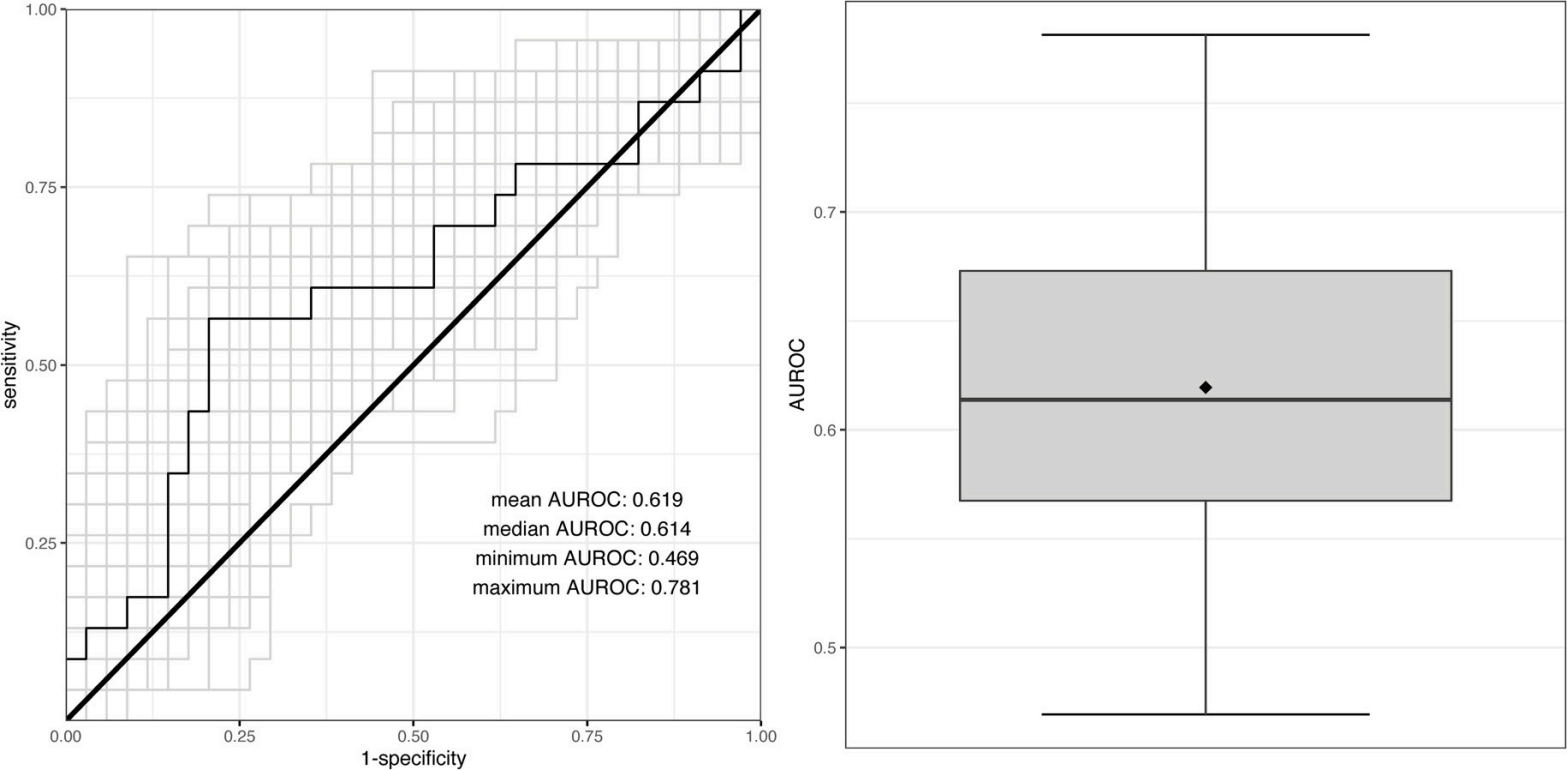
ROCs for all iterations of the modeling procedure to predict CMV DNAemia (left) and the associated AUROCs (right). In the left plot, the ROC that produced the median AUROC is plotted in black, and the remaining ROCs are plotted in grey. In the right boxplot, the median AUROC is shown by the black line, and the mean AUROC is shown by the diamond.

The trained LASSO models were used to predict CMV DNAemia, and the performance of those predictions was measured using the area under a receiver operating characteristic (AUROC). Fig 4 shows the receiver operating characteristics (ROCs, left) curves for all iterations of the modeling procedure, as well as the AUROC values. The mean AUROC was 0.619.

We found that there were marked differences in the predicted probabilities of CMV DNAemia between those transplant recipients who developed CMV DNAemia and those who did not in multiple modeling iterations, (S3.1 Fig in S1 File).

#### Gene expression changes associated with the onset of CMV DNAemia

**Fig 5:**
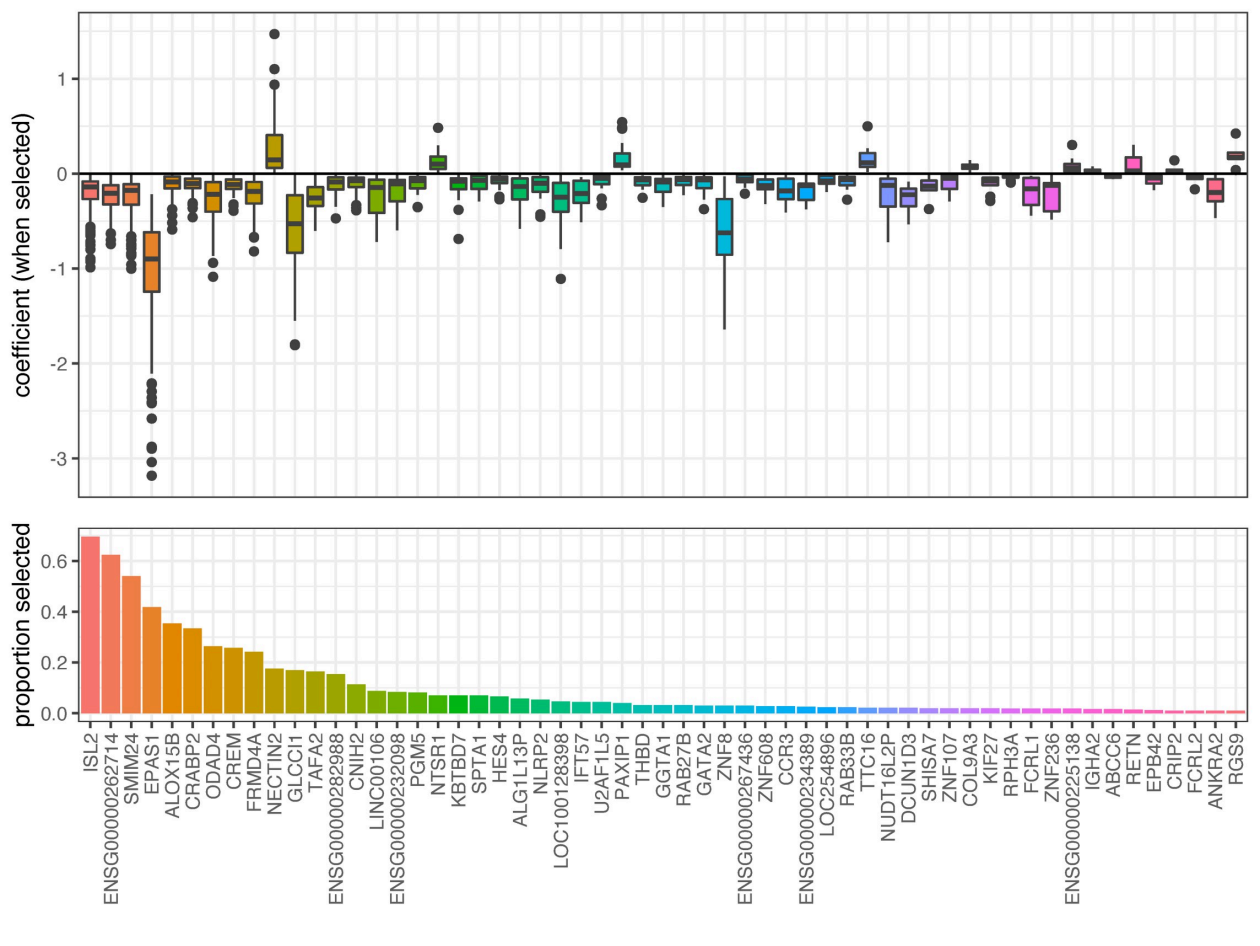
Association analysis for the transplant outcome of clinical CMV (CMV PCR >137 IU/mL) and transcriptomic expression. Boxplots in the upper panel represent the coefficient value of each transcript when selected, and bar plots in the lower panel indicate transcription selection proportion. The transcripts shown are those selected in 1% or more of all training models. Transcripts are labeled with their gene symbol when available or their Ensembl ID. There were no clear trends in coefficient values as the selection proportion decreased.

We next examined the effect of analyzing a stricter definition of CMV DNAemia (indicative of more definite abnormality) by excluding any subject with CMV levels that were less than 137 IU/mL, as an elevated quantitative PCR is more indicative of clinically meaningful CMV infection. However, due to low number of subjects with the outcome of clinical CMV DNAemia (n = 9 subjects), when subclinical CMV subjects were removed from the dataset the smaller sample size precluded the development of significant predictors. Thus, we additionally examined further associations between this outcome and the expression of transcripts with the highest variance. We performed 500 iterations of a procedure in which we subsampled the data in the same manner as the predictive analysis. We used LOOCV to identify a tuning parameter value for LASSO regression that minimized the deviance and then fit a final model for each iteration using that value. Ultimately, 57 transcripts were selected in 1% or more of the fitted models. The majority of the transcripts (47) were consistently negative (Fig 5), and there were no clear trends in coefficient values as the selection proportion decreased.

#### Comparison of gene expression changes in response to CMV DNAemia in an independent dataset

In order to understand how these results compare to published data, we next examined an independent dataset of gene expression changes in response to CMV DNAemia over time in renal transplantation (in the absence of HCV infection) by Ahn, et al [31]. We performed GSEA analysis of this dataset to obtain further nuanced insight and identify overlap between gene pathways identified in our study cohort and this independent cohort of HCV-negative renal transplant recipients. Our study (comparing clinical CMV vs no CMV; all subjects, all timepoints) demonstrated a range of immunologic functions, including adaptive immune response, neutrophil immune response, myeloid leukocyte activation, lymphocyte differentiation, T cell differentiation, and T cell activation. Ahn, et al., showed instead strong expression of specific antiviral responses, including type I interferon signaling, defense response to virus, and negative regulation of viral genome replication–this follows, as that study design was focused on immune responses and sample collection during actual active CMV disease.

### Gene expression differences are less distinct between transplant recipients who develop allograft rejection and those who do not

Given fixed sample collection times, samples were often not available at or near the time of the event of interest (i.e., rejection). The median time from sample collection to rejection (for samples collected prior to the outcome only), was -103 days (IQR -79 to -197)–for controls this was -182 days (IQR -106 to -244). For the HCV cohort, this was -91 days (IQR -47 to -146) (p = 0.224).

To further understand the relationship between sequelae of HCV infection and rejection, we performed univariate analyses of subjects with allograft rejection compared to controls, both in the presence and absence of HCV. To assess for the presence of conserved but persistently dysregulated transcriptomic signals that begin prior to rejection and do not resolve after treatment, we first grouped differential gene expression data in all subjects at all timepoints (all rejection vs no rejection). In the differential expression analysis of all subjects at all timepoints, no genes were significantly up or downregulated in response to allograft rejection. When transplant recipients with rejection were divided by HCV status, differential expression analysis of the control population revealed that 6 genes were significantly upregulated and none were downregulated (by FDR-adjusted p-value <0.05) in response to allograft rejection (including HDAC10, TNNT3, EIF4EBP1, CLEC11A, TMEM205). In the HCV group, only 1 gene was significantly downregulated, cystatin F (CST7), while no genes were upregulated. Upregulation of this gene has been previously identified as a biomarker of acute inflammation [32]. When the HCV cohort was further divided by timing of sample collection, CST7 was noted to be one of the top downregulated genes at the acute and 3- month timepoint, though not at the 6-month timepoint. However, at all individual timepoints, gene expression was not significant.

On univariate comparison of allograft rejection compared to no rejection in the HCV cohort alone, we found that the most quantitatively upregulated genes included multiple immunoglobulin markers, including IGHA2, IGKV3, IGHG1, IGHA1, IGLC2, IGHG2, IGKC. These immunoglobulin genes were not as notably expressed at the acute timepoint but were most strongly expressed at the 3-month timepoint (IGHA2, IGLC3, IGHG1, IGHG2, IGLC2, IGHA1, IGKV3-20, IGHM) with maximum log_2_FC of 3.95, followed by the 6-month timepoint (IGHA2, IGHG1, IGHA1, IGHG2) with maximum log_2_FC of 2.85. Interestingly, these changes appear to be specific to HCV-infected individuals, as they were not seen when examining rejection in the HCV-negative cohort.

#### Gene expression changes associated with the onset of allograft rejection

**Fig 6:**
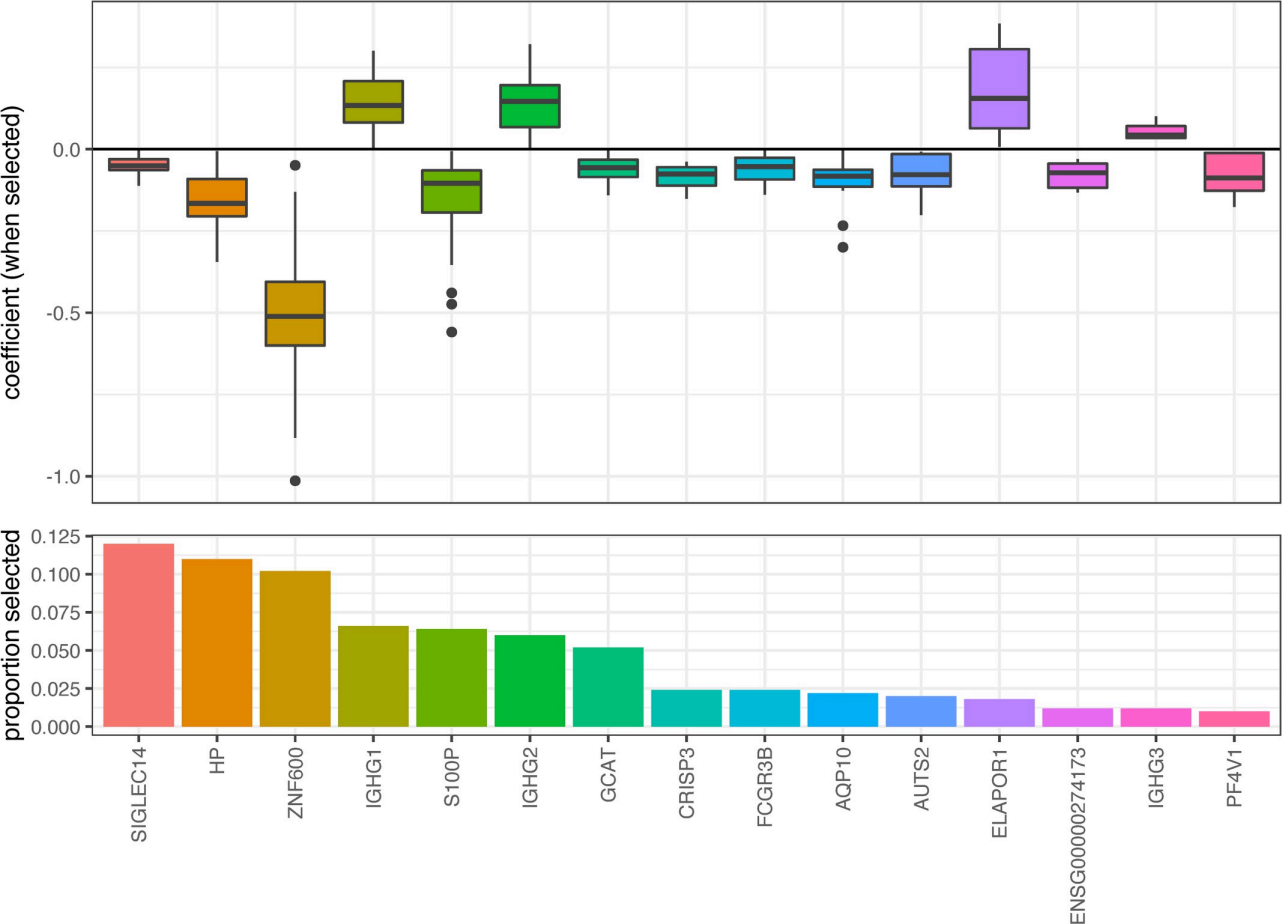
Genes selected in LASSO regression analysis of allograft rejection. Boxplots in the upper panel represent the coefficient value of each transcript and bar plots in the lower panel indicate transcription selection proportion. There are no clear trends in coefficient values as the selection proportion decreases. Genes selected included those involved in immune system activation and hemostasis.

In our predictive analysis the data were subset, where the majority were used to train models in which we constructed relationships between the expression data and future allograft rejection. Each trained model was then used to predict a single future occurrence of allograft rejection. Attempts at predicting future allograft rejection were largely unsuccessful though, in part due to the low proportion of patients that experienced rejection, causing predictions of rejection to be consistently biased (Section 4 in S1 File). The analytic approach shifted instead to an association analysis, in which all of the available data were used to identify relationships between gene expression and future allograft rejection. Of the 1,000 potential transcripts selected, fifteen were selected in 1% or more of the fitted models. The coefficients for 11 of the 15 were consistently negative, although the effects were small in magnitude (Fig 6). These genes include those involved in immunoglobulin and immune system activation (IGHG1, IGHG2, IGHG3, FCGR3B) as well as hemostasis (HP, PF4V1).

#### Comparison of gene expression changes in response to allograft rejection in an independent dataset

We next compared our findings to an independent gene expression dataset from a published transcriptomic signature of active rejection in renal transplantation (in the absence of HCV infection) by Zhang, et al [18]. On differential expression analysis, when we compared all samples from our cohort (rejection vs no rejection; regardless of timing) there was an overlap with only 32 of the genes described by Zhang, et al., in the discovery cohort and only 57 genes with the validation cohort (obtained during acute, active episodes of rejection).

At the pathway level, similar results were seen. GSEA analysis of HCV-NAAT-positive patients with rejection (regardless of sample timing), demonstrated genes involved in the immune pathways, including ‘immunoglobulin complex,’ ‘humoral response,’ ‘B cell mediated immunity,’ ‘phagocytosis,’ ‘defense response to virus,’ and ‘complement activation.’ However, this was distinct from the GSEA analysis in the Zhang, et al., study where involved genes were more consistent with routine cellular functions, as opposed to specialized immunologic functions.

We next applied the transcriptomic signature of subclinical rejection from the Zhang, et al., paper to our HCV allograft dataset (using only samples obtained prior to the rejection event). However, when the independent 17-gene signature of rejection was applied to our data, the signature was unable to clearly differentiate between allograft rejection and controls in both the uninfected donor and HCV donor datasets (S5.1 Fig in S1 File).

## Discussion

While HCV-infected organ transplantation has become an increasingly common practice in clinical medicine, there remain concerns about the impact of acute HCV viremia at the time of transplantation and the downstream implications of this in clinical practice. This work represents the first examination of transcriptomic responses in circulating cells of the organ recipient in the setting of HCV-transplantation and includes sequential timepoint sampling during the time of acute HCV viremia and subsequent antiviral treatment.

In the setting of transplantation from HCV-NAAT-positive donors, we found that acute HCV triggers a profound and robust transcriptomic response in renal transplant recipients. This primarily consists of genes known to be heavily involved in the antiviral response, including interferon-related genes and OASLs. Thirteen of the 109 genes differentially expressed in response to HCV infection were also included in a previously published pan-viral response [33] (Table 2)–including interferon-induced proteins, oligoadenylate synthetases, and serpin family G. Interferons comprise a set of cytokines that impact viral replication, and pegylated interferon was previously a mainstay of HCV antiviral therapy before the development of direct-acting antivirals [34]. OASL is a known component of the antiviral response, impacting viral replication, though may also have pro-viral and biomarker roles [35]. Thus, this gene overlap is not surprising, as these are genes known to be involved in the host immune defense response to viral infections.

**Table 2:** Overlap of HCV genes with the pan-viral signature.

DDX58
HERC5
IFI44
IFI44
IFI44L
IFIT2
IFIT3
ISG15
OAS3
OASL
RSAD2
SERPING1
XAF1

However, it is important to note that this canonical antiviral response exists even in the presence of systemic immunosuppression. This is potentially concerning in the setting of clinical transplantation because it demonstrates that a strong inflammatory response develops rapidly post-transplant, which could lead to downstream dysregulated immunity if left untreated. This parallels the clinical literature that is suggestive of increased adverse outcomes after HCV transplant with delayed antiviral initiation [6, 7]. Though our study did not show discernable increased rates of either CMV reactivation or acute rejection, this may have been limited by small overall study sample size and lack of long-term follow-up. Prior studies have also demonstrated evidence of viral infection inducing adverse clinical outcomes in transplant recipients, particularly rejection [36–38]. This argues for earlier initiation of antiviral therapy to treat and cure HCV and shorten this period of sustained inflammation (pre-emptive rather than reactive). Fortunately, in our data, these same inflammatory responses were no longer significantly expressed at 3- and 6- months post-transplant (after antiviral initiation and completion), indicating that appropriate antiviral therapy and subsequent aviremia improve and resolve this dysregulated signaling.

We also found that there are differences in the gene expression patterns between those transplant recipients who developed CMV DNAemia and those who did not. Though these gene expression results were not statistically significant, the results are quantitatively large, biologically plausible, and include notable overlap with the published literature on gene expression changes in response to CMV infection in renal transplant recipients [31], including genes involved in immune activation and T cell signaling. This may prove to be a further indicator that HCV treatment should be started earlier in the post-transplant course to reduce the presence of this dysregulated signaling and more clearly pick up additional signals predictive of adverse outcomes. However, the relatively small sample size in this study precludes the assigning of statistical significance to these results and identifying a predictive signature for CMV DNAemia in the post-transplant setting. It should also be noted that none of the patients in this study had evidence of documented tissue-invasive CMV infection, and thus likely had milder ‘illness’ which could be associated with less immune stimulation. Additionally, as the samples were collected at pre-determined designated timepoints, and not collected immediately at or prior to a CMV DNAemia event, the delay between sample collection and the clinical outcome (median of -48 days) could impact our ability to identify CMV-associated immune signals. Furthermore, it could be that CMV-associated signal is overwhelmed by the vigorous viral signal from HCV infection that we have shown to be present in the acute setting.

To provide further validation of the findings with CMV, we compared our data to an independent CMV DNAemia gene expression dataset in kidney transplant recipients (in the absence of HCV) [31]. There was overlap of over 300 genes between the two datasets demonstrating that some similar CMV signals were present in both studies. However, the immune response pathways revealed in the HCV dataset were broader in activity and dealt with generalized immune activation and inflammation of multiple components of the immune system compared to the Ahn, et al. study, in which the immune pathways were more focused on antiviral responses. Again, these differences are likely due to variances in timing between sample collection and clinical event between the two studies, as the Ahn study design was focused on immune responses and sample collection during actual active CMV DNAemia, rather than predicting future events.

For allograft rejection, gene expression differences were not as distinct between the HCV and control cohorts. We were not able to identify notable differences in gene expression levels between those transplant recipients who developed allograft rejection and those who did not, again limiting our ability to identify a predictive signature for this clinical outcome. This is likely due to the variable and often large time between sample collection and rejection event (median -103 days) and may also be impacted by the relatively small sample size. Within the HCV cohort, only one gene was significantly downregulated at early post-transplant timepoints between those who would later develop rejection and those who would not, cystatin F (CST7), while no genes were upregulated. Upregulation of this gene has been previously identified as a biomarker of acute inflammation [32]. One hypothesis as to why this gene would be downregulated in such a circumstance may be due to feedback mechanisms, or effects of systemic immunosuppression.

When examining the magnitude of gene expression changes in the setting of rejection compared to no rejection in the HCV cohort, we found that the most upregulated genes included multiple immunoglobulin markers, including IGHA2, IGKV3, IGHG1, IGHA1, IGLC2, IGHG2, IGKC. When examining this subgroup by individual timepoints, these immunoglobulin genes were not as notably expressed at the acute timepoint, but were most strongly expressed at the 3-month timepoint, followed by the 6-month timepoint. This aligns with the known delay from transplant to rejection, particularly of antibody-mediated responses in the setting of *de novo* DSA production. These immunoglobulin genes were not as notably expressed in the HCV-negative cohort, suggesting a role of HCV infection in progressing this pathologic process. This suggests that the presence of HCV infection may trigger B-cell focused hyperactivation, leading to an environment where maladaptive immunologic outcomes, such as DSA production, may occur. Unfortunately, our study did not analyze specific B cell subsets that were exhibiting these changes. Additional studies (including looking at larger HCV datasets or later timepoints) are necessary to determine the target specificity of those B cells undergoing hyperactivation.

We attempted to validate these rejection data with an external gene expression dataset of renal transplant recipients [18]. However, when the Zhang, et al. gene signature was applied to our dataset, it was not predictive of allograft rejection in either the control or HCV samples. This may be due to differences present in the design of the two studies. In the Zhang, et al. study, there were no HCV-infected subjects included. Additionally, there were differences in clinical outcomes between the two studies, particularly overt clinical rejection in our study group (biopsy-proven and/or DSA production) compared to biopsy-proven subclinical rejection in the Zhang, et al. study. Furthermore, samples in the Zhang, et al. study were obtained at the same time as the biopsies that confirmed rejection (rather than prior) and are thus more indicative of signals of active rejection, rather than a predictive signature of future events. Our data show that diagnostic signatures of acute events do not seem to offer strong prediction when there is more prolonged time from event to sampling. Evaluation of whether true prediction of future rejection is possible will require a larger study.

## Conclusion

In conclusion, this work reveals novel components of the character and timing of immune responses to HCV transplantation as seen through the lens of transcriptional biomarkers in circulating leukocytes. We found that HCV infection produces a robust inflammatory response in the acute setting after transplant. This could prove to be maladaptive given the strong early signal despite the presence of systemic immunosuppression and association with adverse clinical outcomes. Gene expression differences are also seen in response to CMV DNAemia, although these findings will need to be studied in larger cohorts. The feasibility of this signal as a predictive clinical biomarker appears to be clouded by the additional overwhelming HCV response. Encouragingly, this signal improves and even resolves with HCV treatment and subsequent aviremia, supporting the practice of early HCV treatment.

## Supporting information

S1 File (DOCX)

## Abbreviations

AUROC: area under a receiver operating characteristic
BP: biological process
C: Celsius
CC: cellular component
CMV: cytomegalovirus
CPM: counts per million
CST7: cystatin F
D: donor
DEA: differential expression analysis
DSA: donor specific antibody
DUMC: Duke University Medical Center
FDR: false discovery rate
GO: gene ontology
GSEA: gene set enrichment analysis
HCV: hepatitis C virus
HSV: herpes simplex virus
IFIT: interferon-induced proteins
IQR: interquartile range
ISGs: interferon stimulated genes
IRB: institutional review board
IU: international units
LASSO: least absolute shrinkage and selection operator
Log_2_FC: log_2_ fold change
LOOCV: leave-one-out cross-validation
MF: molecular function
mL: milliliters
OASL: oligoadenylate synthetase-like
NAAT: positive: nucleic acid amplification test-positive
PCR: polymerase chain reaction
R: recipient
ROC: receiver operating characteristic
SD: standard deviation
STAR: spliced transcripts alignment to a reference
SVR12: sustained virologic response 12 weeks after HCV treatment
TMM: trimmed mean of M-values
