# Supplementary material for: Transcriptional responses define dysregulated immune activation in Hepatitis C (HCV)-naïve recipients of HCV-infected donor kidneys
Source: PLoS One. 2023 Jan 26;18(1):e0280602. doi: 10.1371/journal.pone.0280602 (PMC9879532; doi:10.1371/journal.pone.0280602)
Supplement: S1 File — (DOCX) [file pone.0280602.s001.docx]

**Supplemental Material**

Section 1: Differential Expression Models

**S1 Table. Differential Expression Models**

| Covariate(s) | Samples  (Hepatitis C/Control/all) | Timepoint(s) |
| --- | --- | --- |
| phenotype | all | all |
|  | all | acute |
|  | all | 3 months |
|  | all | 6 months |
| CMV | all | all |
|  | all | acute |
|  | all | 3 months |
|  | all | 6 months |
|  | Control | all |
|  | Control | acute |
|  | Control | 3 months |
|  | Control | 6 months |
|  | Hepatitis C | all |
|  | Hepatitis C | acute |
|  | Hepatitis C | 3 months |
|  | Hepatitis C | 6 months |
| CMV+phenotype | all | all |
|  | all | acute |
|  | all | 3 months |
|  | all | 6 months |
| rejection | all | all |
|  | all | acute |
|  | all | 3 months |
|  | all | 6 months |
|  | Control | all |
|  | Control | acute |
|  | Control | 3 months |
|  | Control | 6 months |
|  | Hepatitis C | all |
|  | Hepatitis C | acute |
|  | Hepatitis C | 3 months |
|  | Hepatitis C | 6 months |
| rejection+phenotype | all | all |
|  | all | acute |
|  | all | 3 months |
|  | all | 6 months |

Section 2: Clinical data of CMV DNAemia and rejection:

**S2 Table. Clinical Data of CMV DNAemia**

|  | **CMV DNAemia** | | | | | | |
| --- | --- | --- | --- | --- | --- | --- | --- |
| **Phenotype** | | **CMV Serostatus**  **(D/R)*** | **Timing**  **(PTD)**** | **Peak**  **(IU/mL,**  **log IU/mL)** | **Duration**  **(Days)** | **Therapy Initiation (PTD)**** | **Therapy Duration (Days)** |
| **Control** | | D+/R- | 199 | 59,700 (4.8) | 47 | 200 | 77 |
|  |  | D+/R+ | 65 | 4,730 (3.7) | 33 | 83 | 25 |
|  |  | D+/R+ | 58 | 3,414 (3.5) | 33 | 60 | 42 |
|  |  | D+/R+ | 69 | 1374 (3.1) | 38 | 79 | 131 |
|  |  | D+/R- | 102 | 1,250 (3.1) | 36 | 109 | 35 |
|  |  | D+/R+ | 23 | <137 (<2.1) | 15 | NA*** | NA |
|  |  | D+/R+ | 15 | <137 (<2.1) | 23 | NA | NA |
|  |  | D+/R+ | 16 | <137 (<2.1) | 13 | NA | NA |
|  |  | D+/R+ | 17 | <137 (<2.1) | 14 | NA | NA |
|  |  | D-/R+ | 0 | <137 (<2.1) | 18 | NA | NA |
|  |  | D-/R+ | 0 | <137 (<2.1) | 9 | NA | NA |
|  |  | D+/R- | 58 | <137 (<2.1) | 86 | NA | NA |
|  |  | D-/R+ | 13 | <200 | 7 | NA | NA |
|  |  | D+/R+ | 24 | <200 | 13 | NA | NA |
|  |  | D+/R+ | 64 | <200 | 7 | NA | NA |
| **HCV** | | D-/R+ | 26 | 783 (2.9) | 30 | 27 | 70 |
|  |  | D+/R+ | 15 | 411 (2.6) | 23 | 20 | 39 |
|  |  | D+/R+ | 230 | 394 (2.6) | 20 | 235 | 42 |
|  |  | D+/R+ | 36 | 280 (2.4) | 16 | 39 | 70 |
|  |  | D+/R+ | 63 | <137 (<2.1) | 21 | 65 | 21 |
|  |  | D+/R+ | 47 | <137 (<2.1) | 14 | NA | NA |
|  |  | D-/R+ | 11 | <137 (<2.1) | 5 | NA | NA |
|  |  | D-/R+ | 145 | <137 (<2.1) | 21 | 147 | 140 |
|  |  | D+/R+ | 308 | <137 (<2.1) | 14 | NA | NA |
|  |  | D+/R- | 22 | <137 (<2.1) | 11 | NA | NA |
|  |  | D-/R+ | 18 | <137 (<2.1) | 14 | NA | NA |
|  |  | D+/R+ | 107 | <227 (<2.4) | 14 | NA | NA |
|  |  | D+/R+ | 63 | <200 (<2.3) | 5 | NA | NA |
|  |  | D-/R+ | 279 | 58 (<1.8) | 24 | NA | NA |

*D/R=donor/recipient

**PTD=Post-Transplant Day

***NA=Not Applicable

**S3 Table. Clinical Data of Antibody Mediated Rejection**

| **Antibody Mediated Rejection** | | |
| --- | --- | --- |
| **Phenotype** | **Nature** | **Strength (MFI)*** |
| Control | CII DQB1*06:01 | 1785 |
|  | CII DQA1*05:05, CII DQ7 | 12842, 13760 |
| HCV | CII DQA1*01:02, CII DQ6 | 2359, 2956 |
|  | CI A23, CII DR53 | 7000, 8793 |

*MFI=Mean Fluorescence Intensity

**S4 Table. Clinical Data of Acute Cellular Rejection**

| **Acute Cellular Rejection** | |
| --- | --- |
| **Phenotype** | **Banff Grade** |
| Control | Borderline inflammation suspicious for T cell mediated rejection |
|  | Borderline inflammation suspicious for T cell mediated rejection |
|  | Borderline inflammation suspicious for T cell mediated rejection |
|  | Cardiac Biopsy (heart-kidney transplant): Focal mild acute cellular allograft rejection; (ISHLT Grade 1R; 1990 Grade 1A) |
| HCV | Banff grade IA |
|  | Banff grade IB |
|  | Banff grade IIA |
|  | Banff grade IIB |

Section 3: Gene expression changes predictive of CMV DNAemia

**
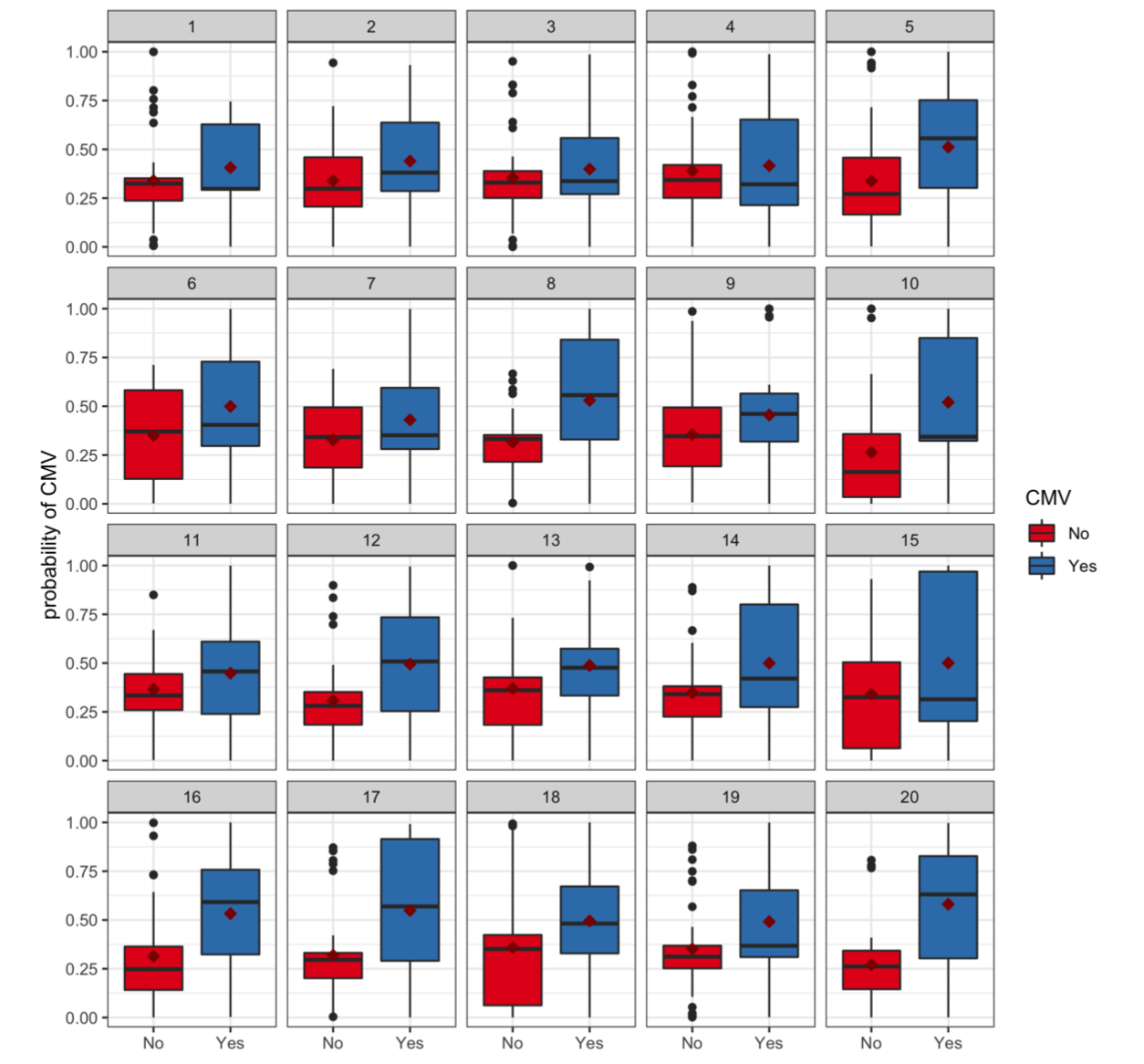
**

**S3.1 Fig. Predicted probabilities of CMV DNAemia for the first 20 (out of 100) modeling iterations.** Each subplot compares the predicted probabilities for patients that experienced CMV DNAemia (Yes) to those that did not (No). The median is shown in a thick black line, and the mean is shown in a red diamond. For many of the models (particularly 8, 10, 16, 17, and 20), there is a marked difference in predicted probabilities between those recipients with CMV DNAemia and those without.

Section 4: Gene expression changes predictive of allograft rejection

**Methods**

To predict allograft rejection, a procedure nearly identical to that used to predict cytomegalovirus (CMV) DNAemia was used. The 1,000 transcripts with the highest variance were used for modeling. Each model fit contained dummy variables for the RNA extraction batches. The same subsampling and cross-validation (CV) procedure described for predicting (CMV) DNAemia was used, with only a slight modification. Briefly, 100 iterations of the following procedure were used. For patients that experienced allograft rejection, the blood sample immediately prior to rejection was used to provide the transcriptomic data. For patients that did not experience allograft rejection, a randomly sampled blood sample was used.

A nested leave-one-out cross-validation (LOOCV) procedure was used to predict allograft rejection. One-at-a-time each observation was held out, and LOOCV, instead of three-fold CV as used for CMV DNAemia, was used on the remaining data to train a least absolute shrinkage and selection operator (LASSO) model that minimized the deviance. The held-out observation was predicted using the model with a regularization parameter value that minimized the deviance.

**Results**

The majority of the models fit across the 100 modeling iterations contained only an intercept and the RNA extraction batch indicators. Out of 6,100 total models fit (61 patients*100 modeling iterations), 5,167, or 84.7%, contained only an intercept and RNA extraction batch indicators.

In these models, predictions made concerning rejection use only the RNA extraction batch-specific intercepts, and they do not incorporate any information that distinguishes between patients that did and did not experience rejection. With small sample sizes and a small proportion of patients that experienced allograft rejection, there is bias inherent in these models that is caused by removing patients for model training purposes. When predicting rejection for patients that experienced rejection, this bias lowers the average probability of experiencing allograft rejection in the training cohort, resulting in the predicted probabilities of rejection in fact being lower for patients that experienced allograft rejection compared with those that did not.

To account for this bias, and to more accurately assess the ability of transcriptomic data to predict allograft rejection, modeling iterations that were heavily influenced by this bias were removed from further analyses in the following way. For each modeling iteration, the proportion of the 61 trained models in which transcripts were selected for inclusion, meaning had more than an intercept and RNA extraction batch dummy variables, was calculated. Modeling iterations in which less than 75% of the models selected transcriptomic information for inclusion were removed from further analyses.

Fourteen out of the 100 modeling iterations had 75% or more training models that included transcriptomic information (Fig 2.1). Fig 2.2 shows the transcripts that were selected in 1% or more of the training models among those fourteen modeling iterations.


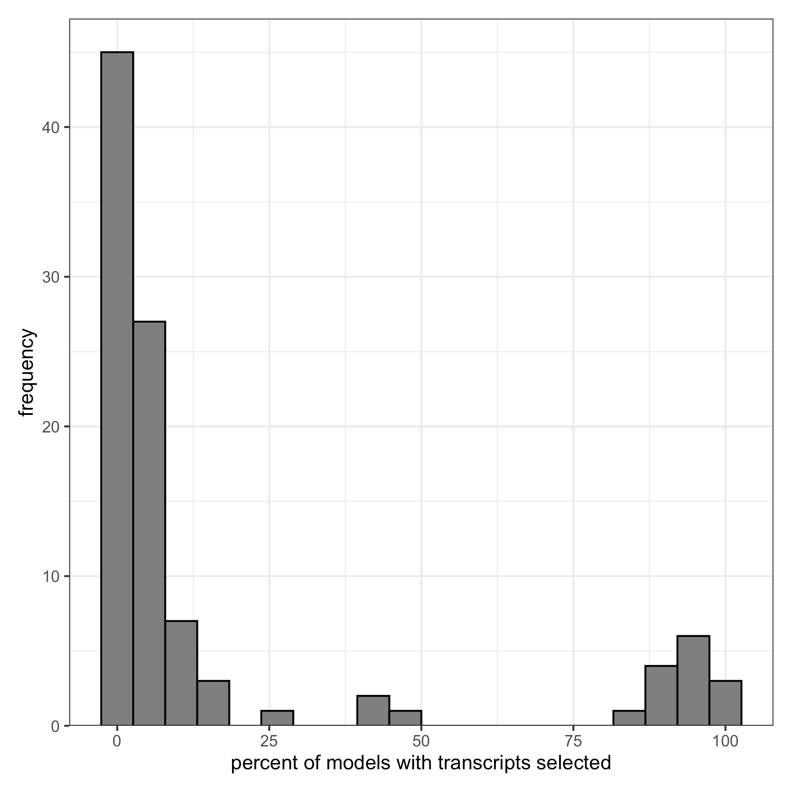


**S4.1 Fig. Percent of training models with transcripts selected.** For each modeling iteration, the percentage of the 61 training models that included transcriptomic information was calculated and plotted as a histogram.


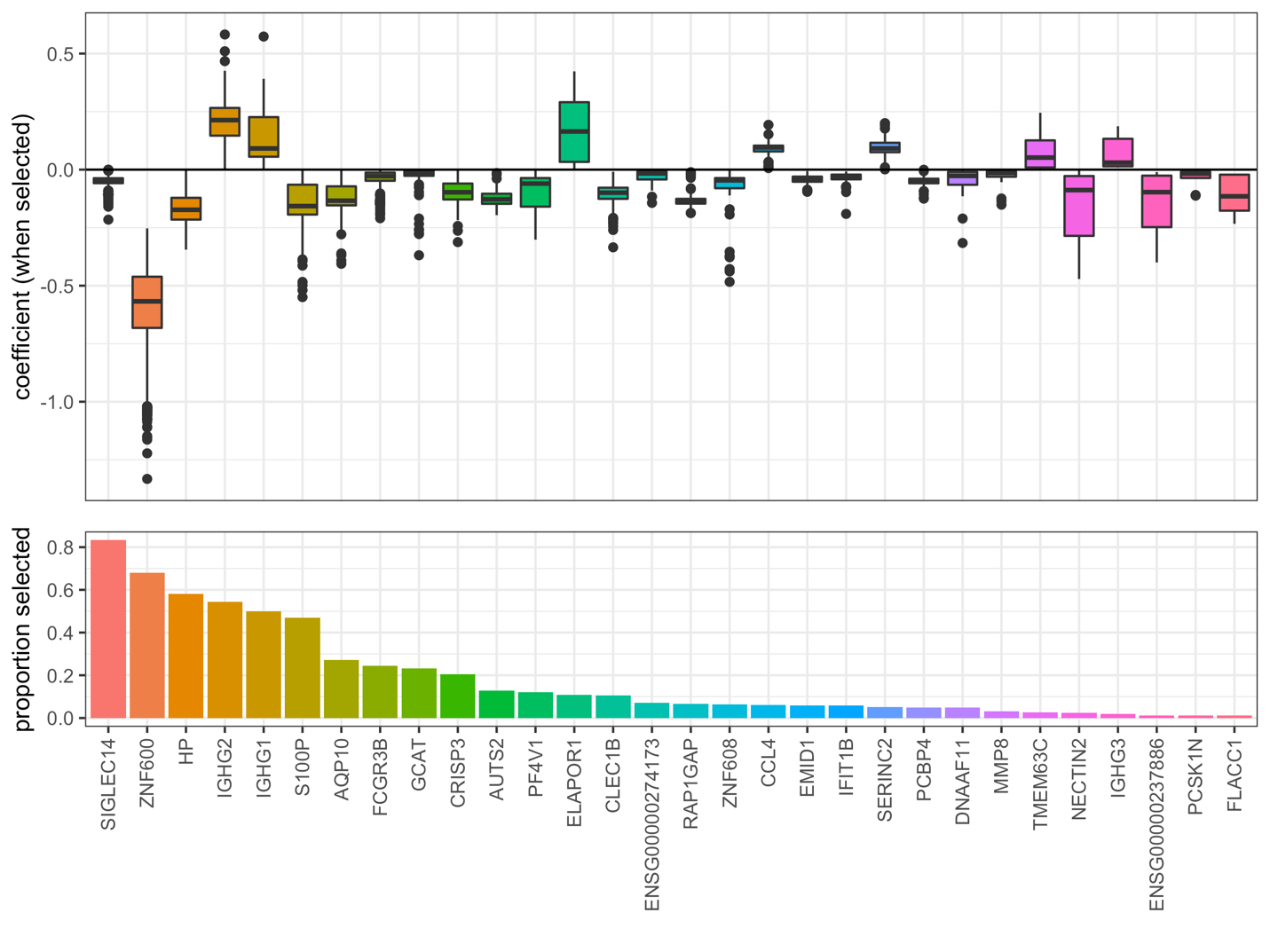


**S4.2 Fig. LASSO regression training results for the prediction of allograft rejection.** Sixty-one LASSO models were trained in each modeling iteration. Shown here are results from the fourteen modeling iterations out of 100 in which 75% or more of the training models selected transcriptomic information. The proportion of these training models in which the corresponding genes and transcripts were selected is shown in bar plots (bottom), and their respective coefficient values are shown in boxplots (top). The transcripts selected in 1% or more of the training models are shown, and they are labeled with their gene symbol when available or the Ensembl ID.

Those fourteen modeling iterations produced areas under a receiver operating characteristic (AUROCs) ranging from 0.449 to 0.698, with a mean of 0.589 and a median of 0.598 (Fig S2.3.). Lowering the elastic net penalty parameter α from 1 (LASSO) to 0.1 resulted in only twelve modeling iterations that exceeded the 75% threshold (data not shown).

**
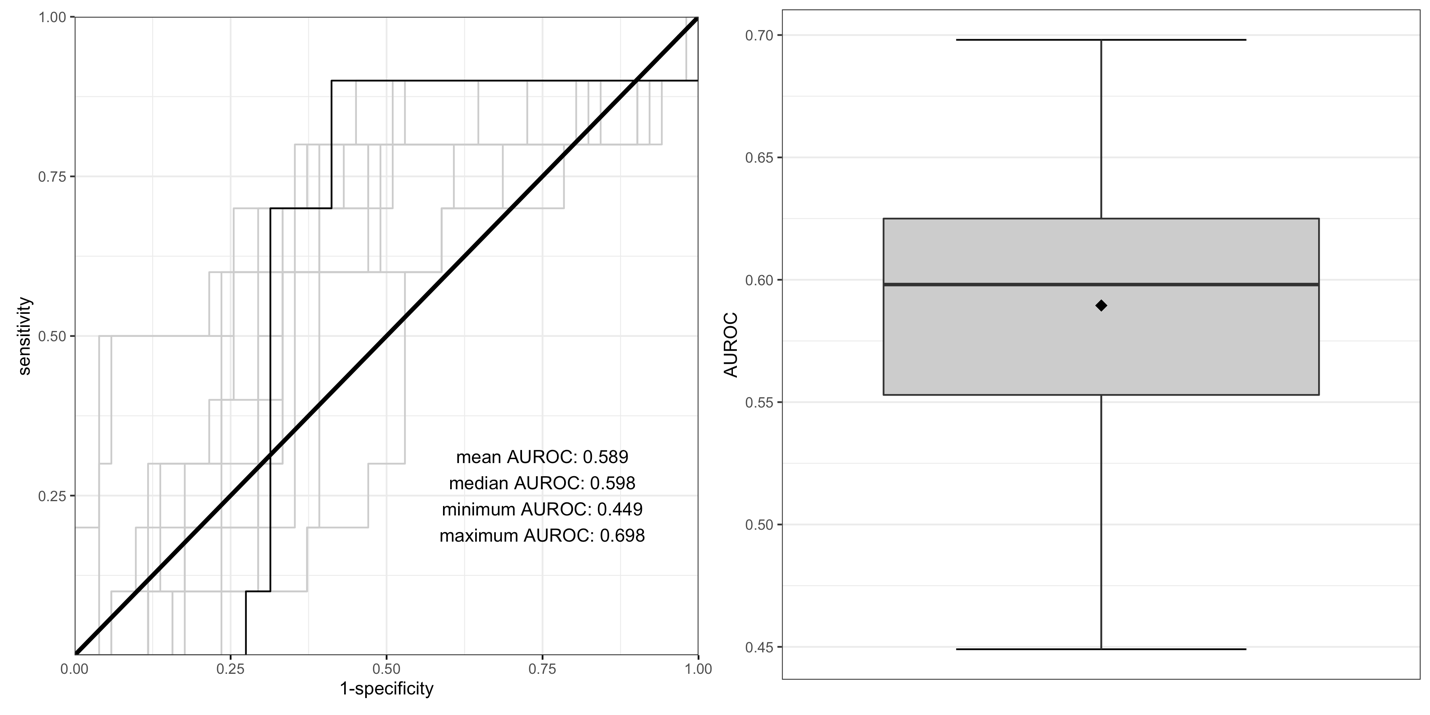
**

**S4.3 Fig. Performance for modeling iterations in which transcriptomic information was frequently selected.** The receiver operating characteristic (ROC, left) and AUROCs (right) are shown for modeling iterations in which at least 75% of the training models selected transcriptomic information for inclusion. The ROC for the model that produced the median AUROC is plotted in black, while the other curves are plotted in grey. In the right boxplot, the median AUROC is shown by the black line, and the mean AUROC is shown by the diamond.


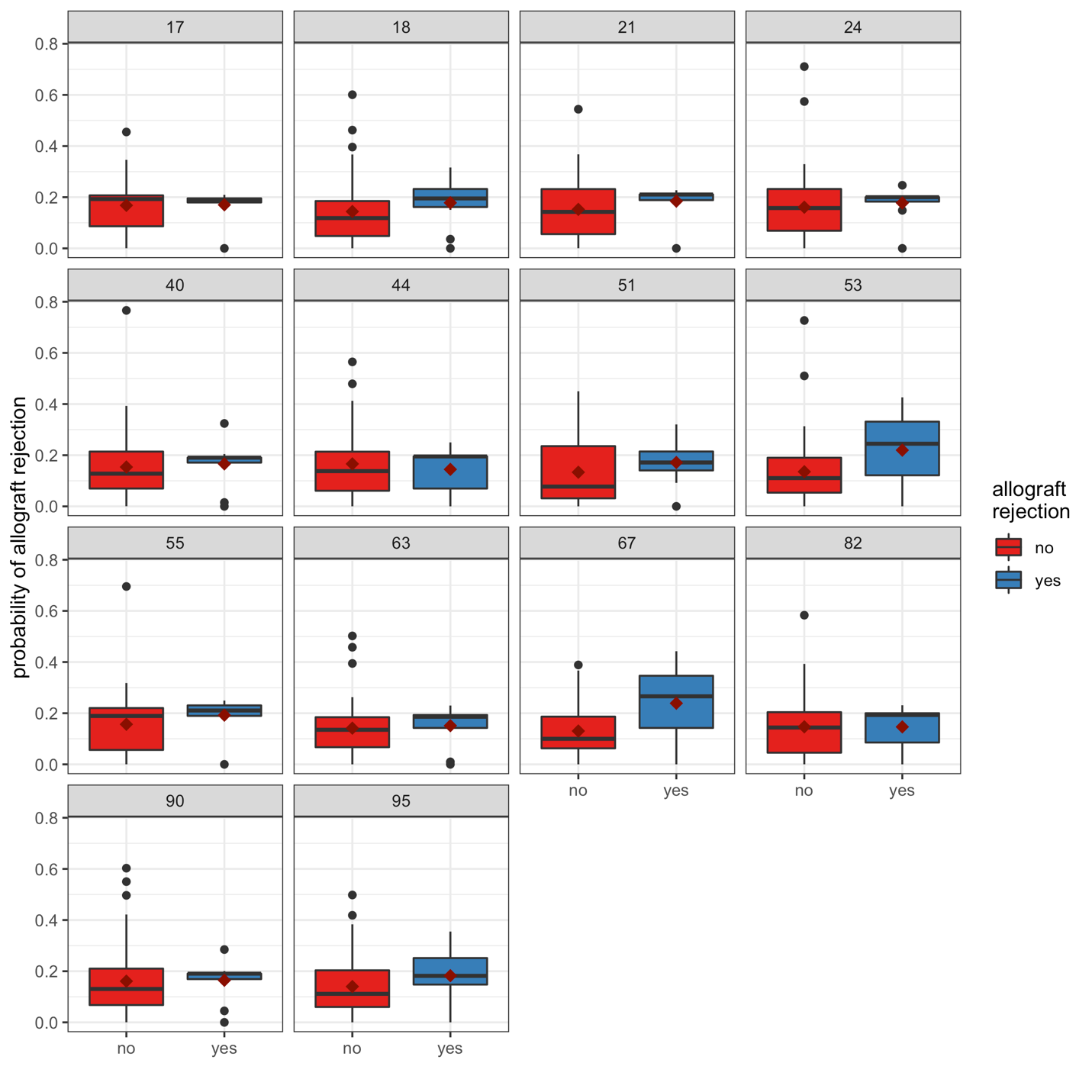


**S4.4 Fig. Predicted probabilities of allograft rejection for the 14 (out of 100) modeling iterations in which 75% or more training models selected transcriptomic information.** Each subplot compares the patients that did (Yes) and did not (No) experience allograft rejection. The median is shown in a thick black line, and the mean is shown in a red diamond.

Section 5: Comparison of gene expression changes in response to allograft rejection in an independent dataset


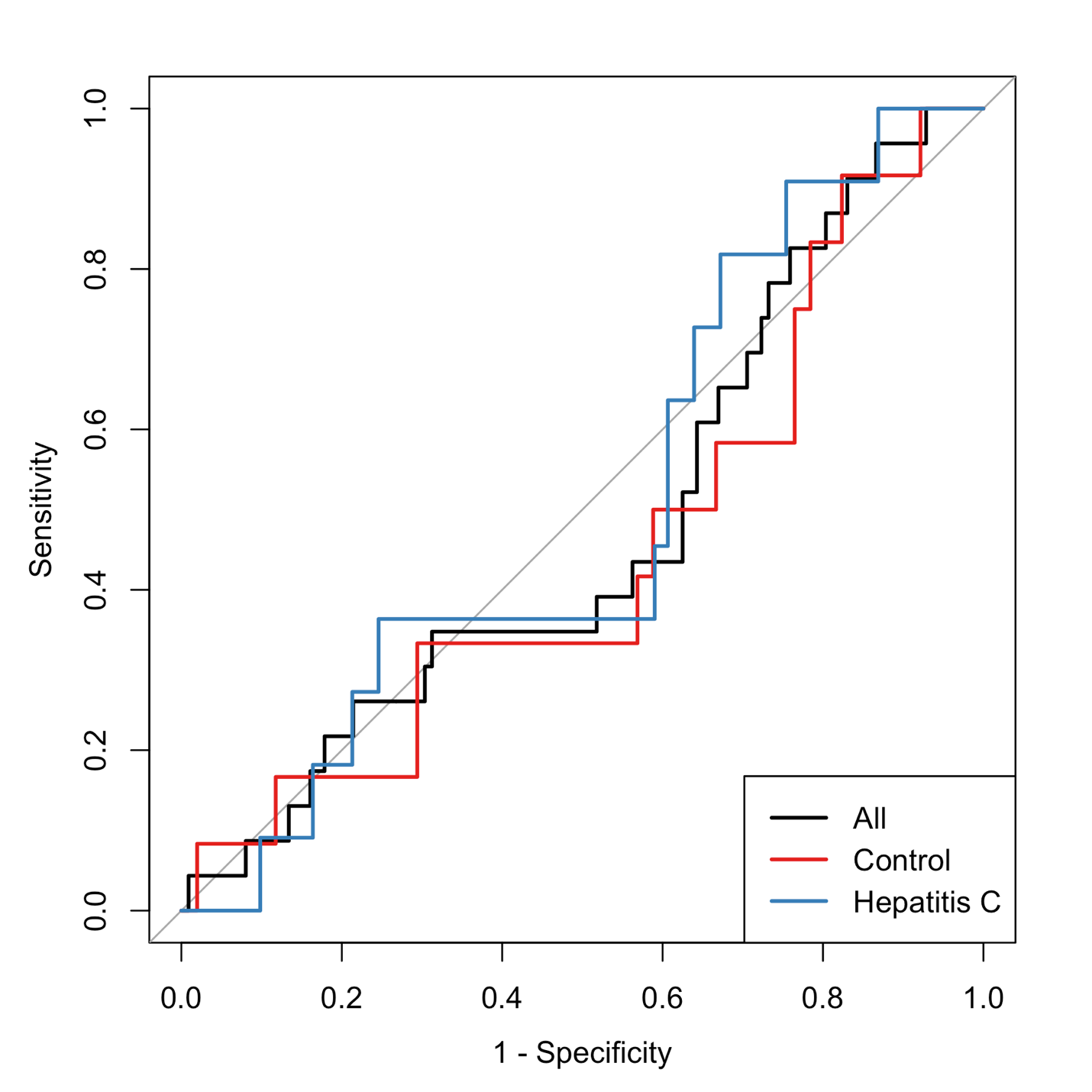


**S 5.1 Fig. Application of Zhang, et al. transcriptomic signature of allograft rejection to HCV dataset.** The signature was unable to clearly differentiate between rejection and no rejection in both the uninfected donor and HCV donor datasets.
